# Motor beta oscillations contribute to the temporal binding effect

**DOI:** 10.1016/j.bandc.2025.106362

**Published:** 2025-09-30

**Authors:** José Luis Ulloa, Roberta Vastano, Ole Jensen, Marcel Brass

**Affiliations:** 1 Programa de Investigación Asociativa (PIA) en Ciencias Cognitivas, Centro de Investigación en Ciencias Cognitivas (CICC), Facultad de Psicología, Universidad de Talca, Chile; 2 Department of Experimental Psychology, Ghent University, Henri-Dunantlaan 2, 9000 Ghent, Belgium; 3 University of Miami, Department of Neurological Surgery, The Miami Project to Cure Paralysis, Miami, FL, USA; 4 Department of Experimental Psychology, University of Oxford, Oxford, OX2 6GG,UK, Oxford Centre for Human Brain Activity, Oxford Centre for Integrative Neuroimaging, Department of Psychiatry, University of Oxford, Oxford, OX3 7JX, UK; 5 Berlin School of Mind and Brain/Department of Psychology, Humboldt-Universität zu Berlin, Germany

**Keywords:** Temporal binding, automatic imitation, EEG, beta oscillations, motor system

## Abstract

Agency, the feeling of controlling one’s actions and their consequences, is closely linked to temporal binding, a phenomenon where the interval between a voluntary action and its outcome is subjectively compressed. While prior research has linked temporal binding to sensorimotor processes, the role of neural oscillations remains unclear. In this study, we combined electroencephalography with an automatic imitation task to examine how trial-by-trial variations in motor-related brain rhythms predict temporal binding. Twenty-eightparticipants performed lifting finger movements in response to visual imperative stimuli. Following each response, they estimated the interval between their action and a subsequent tone. Time-frequency analysis and linear mixed-effects modeling revealed that reduced beta desynchronization predicted stronger temporal binding, independent of action congruency. These results suggest that motor beta oscillations reflects the temporal experience of action-effect coupling, likely reflecting predictive motor processes involved in the construction of voluntary actions.

## 1 Introduction

What marks an action as mine? When I move on purpose, my brain is set for what should happen next, and action and outcome feel closer in time. This time compression, called temporal binding, means that when a voluntary action produces a sensory event, the action and its outcome are judged as nearer in time than they are (Haggard et al., 2002; Moore & Obhi, 2012). Temporal binding is typically measured with Libet-style clock reports, where participants indicate the perceived time of an action or a tone (Libet et al., 1983) or with simple action–outcome interval estimation tasks (Hughes et al., 2013). In time interval estimation tasks, shorter reported intervals (more negative errors) indicate stronger temporal binding (Cravo et al., 2009; Kühn et al., 2013; Wen et al., 2015). An open question, and the focus of this study is what are the brain processes that support this temporal binding between action and its sensory consequences.

Voluntary action engages core elements of motor control that are well placed to shape temporal binding. In internal model of motor control each motor command is accompanied by an efference copy used to anticipate upcoming sensory states—what will be perceived and when (Miall & Wolpert, 1996; Von Holst & Mittelstaedt, 1950; Wolpert et al., 1995). Incoming feedback is compared to this anticipation in a comparator mechanism (Blakemore et al., 2002; Frith et al., 2000). Small mismatches strengthen the sense of causation and compress the perceived action–outcome interval (i.e. temporal binding; Haggard, 2017; Haggard et al., 2002). This computation relies on a sensorimotor circuit: primary motor cortex (M1) and accessory areas such as the supplementary (SMA) and pre-supplementary (pre-SMA) motor areas issue motor commands (Nachev et al., 2008) and distribute efference copies (Crapse & Sommer, 2008; Sommer & Wurtz, 2002) while posterior parietal cortex (including the temporal parietal junction) and superior temporal regions compare predicted with received signals and show sensory attenuation for self-generated input (Blakemore et al., 1998; Houde & Nagarajan, 2011; Waszak et al., 2012).

Evidence from previous studies where temporal binding has been investigated converges on this motor control network. Studies using fMRI or invasive recordings show that temporal binding engage M1, SMA/pre-SMA and parietal and temporal cortices (Kühn et al., 2013; Noel et al., 2025; Seghezzi & Zapparoli, 2020; Wolpe et al., 2014; Zapparoli et al., 2020). Event-related potentials (ERP) studies links stronger temporal binding to greater motor preparation (a more negative readiness potential; Jo et al., 2014) and to reduced sensory responses (attenuated N1) to self-generated outcomes (Poonian et al., 2015). This picture on the neural correlates of temporal binding has been refined by studies focusing on brain rhythms. In an imagery protocol with a robotic grasp, increased temporal binding for actions executed immediately before the grasp was accompanied by stronger beta power suppression over sensorimotor areas (Venot et al., 2024). In addition, visuospatial attention shifts that accompany temporal in binding in a Libet-clock task are associated with occipital alpha desynchronization (Wang et al., 2025). In other studies, larger temporal binding is associated with activity of alpha rhythms in motor circuits (Bertoni et al., 2025). Together, these findings suggest that sensorimotor processes, particularly associated with alpha and beta oscillations, might play a key role in linking voluntary action to its sensory consequences. However, much of this evidence comes from single-case or highly specialized samples (e.g. brain-computer interface users or clinical cohorts), which limit generalizability to everyday voluntary actions and calls for replication. Accordingly, a first aim of our study was to examine whether motor oscillatory activity (in particular in the range of alpha and beta) is associated with temporal binding in healthy adults.

There have been studies investigating how distinct factors modulate temporal binding (e.g. Haering & Kiesel, 2014; Moore et al., 2009; Moreton et al., 2017), yet few have directly tested how motor processes themselves shape this effect. Temporal binding should be strongest when the action a person is required to perform closely matches an action they are observing or that the environment suggests, this is, when an action is performed fluently. We can manipulate this match by introducing a congruency factor. Incongruent actions, where the required and observed actions mismatch, should introduce a conflict. This conflict could disrupt the brain’s ability to successfully predict the outcome of its own movement and increase the cognitive control needed to perform the task, ultimately weakening temporal binding. The imitation–inhibition task offers a standard, trial-wise manipulation of such congruency and reliably engages sensorimotor systems (Brass et al., 2000, 2005, 2009; for a review see Cracco et al., 2018). Indeed, prior work has shown that action fluency in this task can modulate temporal binding: congruent (fluent) relative to incongruent (non-fluent) actions yield stronger temporal binding (Vastano et al., 2017, 2020). Assuming a putative relationship between motor-related brain oscillatory activity and temporal binding, a second aim of our study was to assess whether action congruency (congruent vs. incongruent actions) can modulate this relationship.

How can motor oscillatory processing contribute to temporal binding? Two strong candidates are alpha and beta oscillations. Alpha (8–13 Hz) indexes sensory gating and attention (de Lange et al., 2008; Jensen & Mazaheri, 2010) and, over sensorimotor cortex, mu rhythm engagement reflects mechanisms associated with sensorimotor processing (Foxe & Snyder, 2011; Pineda, 2005). Beta (15–30 Hz) indexes motor state and sensorimotor set, showing pre-movement suppression during command issuance and a post-movement beta rebound (PMBR) during evaluation (Jurkiewicz et al., 2006; Pfurtscheller et al., 2003). Previous studies support the idea that alpha and beta oscillations play a role in temporal binding (see above, Bertoni et al., 2025; Venot et al., 2024; Wang et al., 2025). In addition, other frequency bands such as theta and gamma oscillations may also be associated with changes in temporal binding. Midfrontal theta oscillations (4–8 Hz), linked to conflict monitoring and control (Cavanagh & Frank, 2014; Cohen & Cavanagh, 2011), could be associated with a weakening of binding when control demands rise (e.g., in incongruent actions). Meanwhile, movement-related gamma activity (70–100 Hz), which facilitates precise sensorimotor integration (Cheyne, 2013; Cheyne et al., 2008), might simultaneously signal the need for this control. If present, modulations in theta and gamma frequency bands would suggest a role of control/monitory mechanisms in temporal binding.

In a previous study we coupled an imitation–inhibition manipulation with a standard temporal binding measure and recorded electroencephalographic (EEG) signals. We asked participants to perform a finger lift movement in response to an imperative stimulus and just after they responded they were prompted to estimate the time elapsed between their lifting action and a tone (time estimation task). Here, we re-analyze this dataset to assess the impact of motor-related brain oscillatory activity and action congruency on temporal binding. To respond to our question, we first characterized brain oscillatory activity during participants’ responses. Next, we conducted a linear mixed-effects (LME) model analysis to determine whether brain oscillatory power and congruency (congruent versus incongruent actions) in the imitation-inhibition task predicted temporal binding scores (measured as time estimation errors). If temporal binding is constructed within sensorimotor circuitry, its time course should be reflected in ongoing (trial-by-trial) brain activity.

## 2 Materials & methods

This study used the same dataset reported in our previous manuscript (Vastano et al., 2020). However, the nature of the data analysis differs and none of the neural responses reported here were included in the previous study. In the current study we investigated the congruency effects of automatic imitation on neural oscillations rather than event-related potentials that were the focus of the previous study.

### 2.1 Participants

Twenty-eight participants (age range: 18 to 28 years, mean 23.3 years, 20 females, 8 male) participated in the experiment after giving written informed consent. The study was approved by the local ethical committee of Ghent University and conducted according to the Declaration of Helsinki. All participants were right-handed and had normal or corrected-to-normal vision. All the participants were neurologically and psychiatrically healthy. Participants were paid for their participation.

### 2.2 Stimuli & task

**Figure 1:**
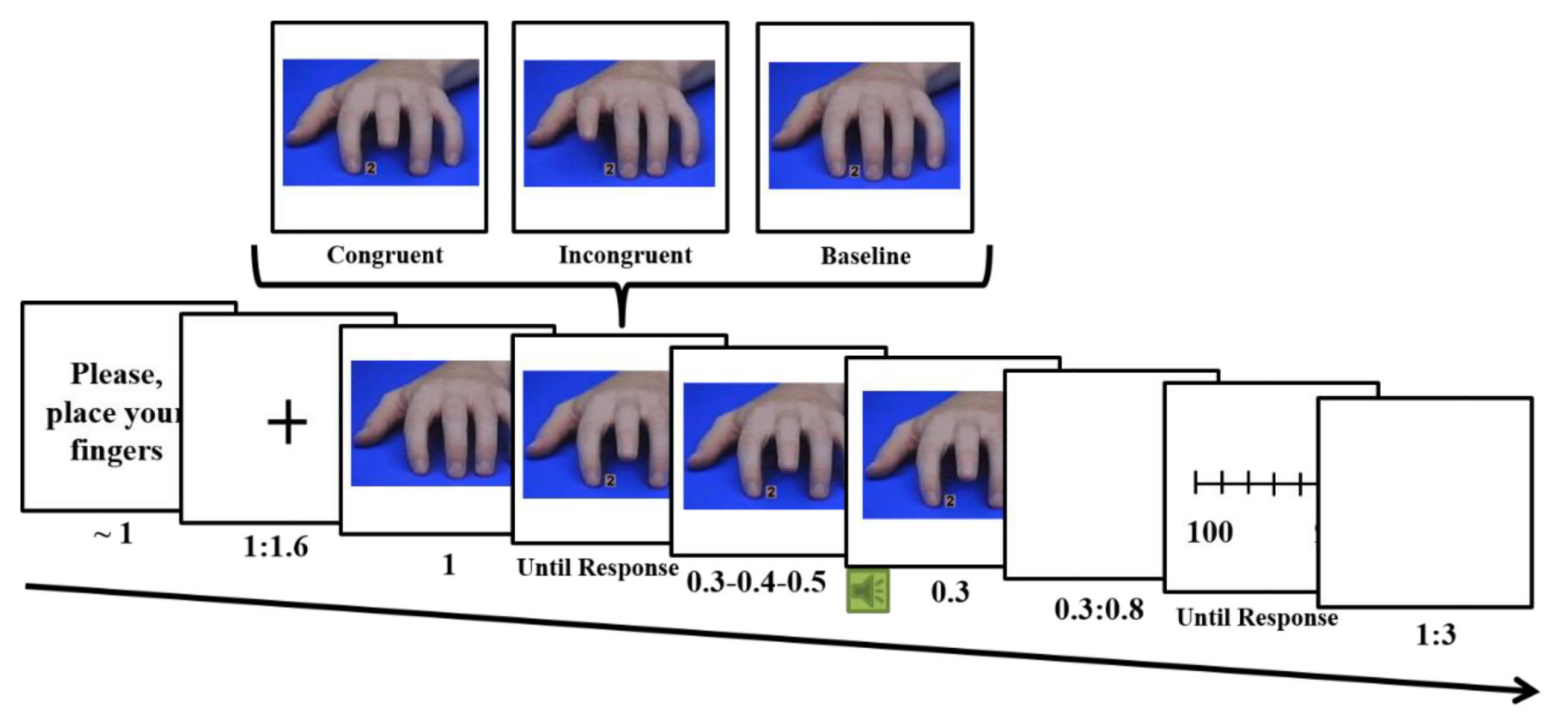
Timeline of a trial. After placing their fingers and fixating a cross, participants responded to a numeric cue displayed between the index and middle finger of a hand (this cue indicated which action participants should do). At the same time the displayed hand moved a finger that could be *Congruent* or *Incongruent* with the participant’ action. A condition where there was no movement of the displayed finger was also included (*Baseline*). After the participant’s response and a random interval (either 0.3, 0.4 or 0.5 s), followed a tone (the action outcome, the speaker in the figure). After a delay (between 0.3-0.6 s) a Visual Analogue Scale (VAS) ranging between 100 and 900 appeared. The participants had to judge on this VAS the time elapsed between their actions and the tone. The difference between the real interval and the estimated interval reflects temporal binding. Finally, after a variable inter-trial interval (between 1-3 s) the next trial started.

The participants were seated in a comfortable armchair in a dim sound-attenuated room at a distance of 60 cm from the computer monitor (refresh rate 60 Hz, dimensions 53 × 30 cm and resolution 1920 × 1080). Visual stimuli were presented on a computer screen and auditory stimuli via headphones. Visual stimuli consisted of images (300 × 200 pixels) of a mirrored right-hand of an actor performing lifting finger movements (see Figure 1). At the start of a trial the message “Please, place your fingers” was displayed. The participants had to hold down the “G” and “H” keys of a Mac keyboard with numeric keypad (MB110Z/B) with their right index and middle fingers respectively. Once the participant placed her/his fingers the sequence continued. A fixation cross was presented between 1000 and 1600 ms and then an image showing a hand in a resting position was displayed for 1000 ms. This was followed by two simultaneous events: a number display (1 or 2 appearing between the two fingers) and the lift of one of the observed fingers (index or middle). Participants were instructed to respond as fast as possible to the ‘1’ by lifting the index finger and to the ‘2’ by lifting the middle finger. If no response was given within a time window of 1400 ms the next trial was presented and the missed trial was recovered. Following the key release and a variable interval (300, 400, or 500 ms) an auditory stimulus (a pure tone at 1000 Hz) was delivered for 300 ms by means of headphones. Next, after a variable interval between 300 and 800 ms a Visual Analogue Scale (VAS) appeared. The VAS ranged between 100 and 900 ms and has marks of 200 ms-intervals. In order to measure temporal binding, participants were asked to estimate the interval of time between their actions (key release) and the ensuing tone using the VAS. For this time estimation task participants used the mouse to point in the VAS. They had a maximum of 5000 ms to answer. Finally, after a variable inter-trial interval (1000, 2000 or 3000 ms) the next trial started. The critical manipulation in this experiment is that the observed finger movements could result in a match or in a mismatch with the instructed finger movement. In congruent (C) trials the participant moves the index finger and sees an index finger movement (and similarly for middle fingers). In incongruent (IC) trials the participant moves the index finger and sees a middle finger movement (or alternatively moves the middle finger and sees an index finger movement). In addition, there was also a condition where the number was displayed but the fingers didn’t move (baseline; B). The experiment consisted of 240 randomized trials: 80 trials for each condition (congruent, incongruent and baseline), each of which was composed by 26-28 trials for each interval (300, 400, and 500 ms). The experiment was divided in 4 smaller blocks of 60 trials each (20 trials in each congruent, incongruent and baseline) to allow participants to rest between blocks. Before the main experiment, the participants were trained for the time estimation task and then were familiarized with the task. For the training, participants listened to two tones separated by 100 or 900 ms and then they were asked to indicate if the time elapsed between the two tones was 100 or 900 ms., for which they received feedback (correct or incorrect response). Participants were trained with 30 randomized trials (15 for each interval). This short training phase aimed at capacitating the participants to discriminate between 100 and 900 ms. Once the instructions were clear the main experiment starts. For the sake of the manipulation in the main experiment the participants were told the interval between their action and the tone was always random between 100 ms and 900 ms. The task was implemented in E-prime 2.0 Professional software (Psychology Software Tools, Pittsburgh, PA). The duration of the whole experiment was about 80 min.

### 2.3 EEG recording & signal processing

Scalp EEG was recorded from 64 active Ag-AgCl electrodes (Biosemi Active-Two, Biosemi, Amsterdam) mounted in an elastic cap according to the international 10–20 setting. The continuous EEG was recorded with a 1024 Hz sampling rate and referenced online to the CSM-DRL ground. Data was recorded in an electrically shielded chamber and electrode offsets were kept between -25 and 25 µV for all electrodes. Additional electrodes in a bipolar montage were applied near the canthi, and above and below the left eye to record the electrooculogram (EOG). EEG data analysis was performed using EEGlab 14.0.0b (Delorme & Makeig, 2004) and Fieldtrip (version 20170503; (Oostenveld et al., 2011) in MATLAB R2014b (The Mathworks, Inc, Natick, MA). The raw EEG data was loaded in EEGlab and filtered off-line.

#### 2.3.1 Artifact rejection

In the first step raw data was filtered between 0.5 and 40 Hz. Non-stereotyped artefacts were cleaned, and bad channels were detected by visual inspection. Bad channels (F6, FC6, T7, P2, and Iz across eight different participants) were interpolated using spherical splines (Perrin et al., 1989). Next, stereotyped artefacts (such as eye movements, eye blinks and muscle tension) were reduced by independent component analysis (ICA) and the SemiAutomatic Selection of Independent Components (SASICA) toolbox (Chaumon et al., 2015), removing no more than 3 components. ICA decompositions were performed separately on each subject over all conditions and then saved. In a parallel step, raw data was not filtered. The same corrections regarding non-stereotyped artefacts and bad electrodes were re-applied to this dataset. Following this, the ICA weights computed before were also re-applied to this dataset. Applying the pre-computed weights allowed to efficiently remove artefacts while retaining all EEG frequency information (Artoni et al., 2017; Oddo et al., 2016; Winkler et al., 2015). Finally, this dataset was re-referenced to the average of all electrodes and visually checked one last time for artefacts. Any portion of data with remnant artefact was eliminated. The final dataset retained in average 66.5 trials (83%) per condition from the original raw data.

### 2.4 EEG analyses

Time-frequency representations of power were estimated in single-trial data in two ways. For frequencies below 40 Hz we used short-time Fourier transform with sliding windows of 500 ms multiplied with a Hanning taper and moving steps of 50 ms. The frequency resolution was 2 Hz. For frequencies above 40 Hz we used a multitaper approach with a window length of 400 ms and frequency smoothing of 20 Hz (i.e. 15 tapers). Power was estimated as the square of the analytic signal z (power = real[z(t)]^2^ + imag[z(t)]^2^) and averaged across trials. Power values at each time-frequency point were normalized by converting to relative change ([Power_task-Power_baseline]/Power_baseline)) to account for power-law scaling of oscillations in different frequency bands. Time-frequency analyses were time-locked to the onset of the subject responses with a -1300 to 1400 ms time-down. Power from -750 to -1000 ms in the pre-stimulus period (when the observed hand stays still) served as the frequency band-specific baseline.

### 2.5 Statistical analyses

**Figure 2:**
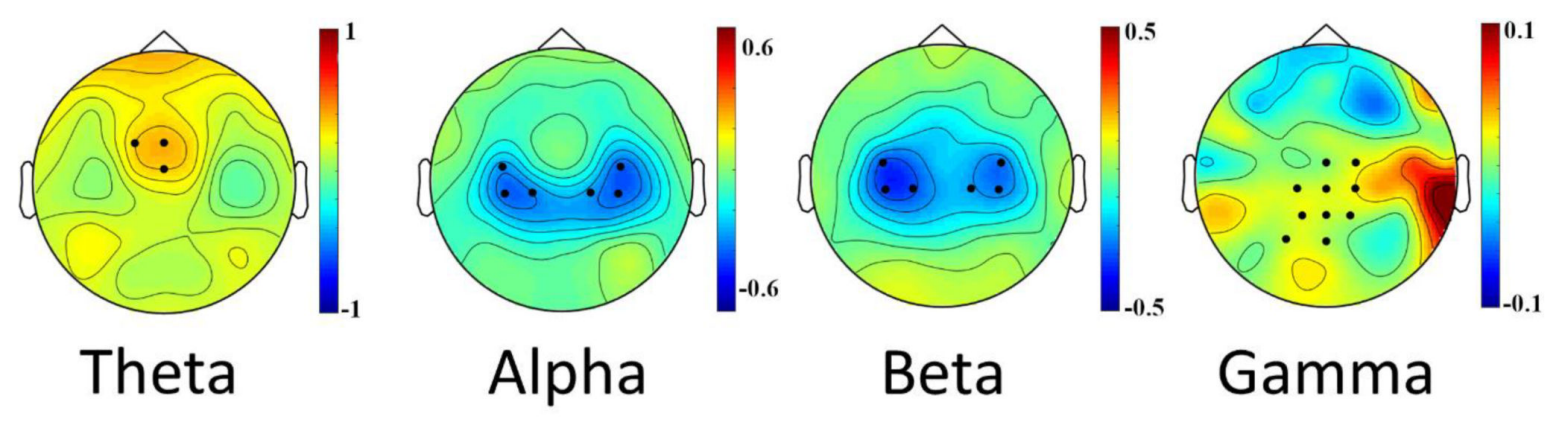
Movements-related changes of neural activity. Topographic distributions of grand-average theta (4-8 Hz), alpha (8-12 Hz), beta (13-30 Hz) and gamma (70-100 Hz) activity (relative to baseline) across different time intervals ranging from -300 to 300 ms around movement onset. A pre-stimulus period, during which the hand remained stationary, served as the baseline. Highlighted electrodes reach statistical activation relative to baseline. The scale represents relative changes in scores.

In the first step, we determined movements-related changes of neural activity. We defined a priori set of frequencies of interest based on previous literature. The range of frequencies of interest consisted of 4-8 Hz for theta, 8-12 Hz for alpha/mu, 15-30 Hz for beta and 70-100 Hz for gamma. Post-movement beta rebound (PMBR) was a theoretically relevant oscillation in our study. We didn’t analyze PMBR because our task timing didn’t allow a clean estimate of beta rebound. In our paradigm the outcome tone occurred 300–500 ms after the key-release, and a VAS prompt for a speeded rating appeared 300–800 ms later. Thus, the post-movement window is rapidly filled with (i) an auditory event and (ii) attentional/motor demands for the rating. Given our analysis epochs the reliable right-edge time was limited, and any PMBR (typically occurring between ~600–1000 ms after movement end and extending beyond 1.5 s; Cheyne, 2013; Gaetz et al., 2010) was contaminated by those events. Exploratory checks indeed did not reveal a robust PMBR across participants, consistent with these constraints. To avoid misinterpretation from a low-SNR, confounded estimate, we restricted our analyses to beta desynchronization during movement response, which our design sampled well. To define movements-related changes of neural activity we averaged response-locked EEG data across all conditions separately for each participant and then submitted to a cluster-based permutation test (Maris & Oostenveld, 2007). No finger movements were observed in the baseline condition; thus, baseline trials were not included in the analysis. The cluster-based permutation test is a procedure that is independent of any condition-specific differences in power and therefore does not introduce any biases into the analysis. We compared a 600-ms window of interest (between -300 to 300 ms relative to response onset) with a baseline window (between -1100 to -500 ms relative to response onset) to determine activities statistically distinct from baseline. The applied statistical procedure controlled the Type I error rate regarding multiple comparisons over the included 64 channels using a clustering approach. For each frequency bin, t-statistics were computed for all electrodes and for each 50 ms time bin within the 600 ms window of interest. An algorithm identified clusters of contiguous electrodes across participants having a threshold below a p-value of 0.05. Subsequently, the cluster-level statistics were defined from the sum of the t-values of the electrodes in a given cluster. The cluster with the maximum sum was used in the test statistics. Type I errors were controlled by evaluating the cluster-level test statistics under the randomization null distribution of the maximum cluster-level statistics. This null distribution was computed by randomly reassigning the data to the conditions (active and baseline window) across multiple participants and subsequently calculating the test statistics for the new set of clusters. A reference distribution of cluster-level t-statistics was created from 1000 random draws. The p-value was estimated according to the proportion of the randomization null distribution exceeding the observed cluster-level test statistic (the Monte Carlo p value). Once these channels and times were identified, we selected active electrodes with the highest activity (above 20%) for posterior analyses. Activities significantly different from baseline were restricted to electrodes FCz, Cz and FC1 between -200 to 200 ms relative to response for theta; to electrodes C3, CP3 and CP1 between -300 to 300 ms relative to response for both mu and beta (in a left cluster); to electrodes C4, CP4 and CP4 between -300 to 300 ms relative to response for both mu and beta (in a right cluster); and to electrodes Cz, C2, CP1, CPz, CP2, P1, P2, Pz, PO3, POz between 50 to 300 ms relative to response for gamma. Visual inspection of the averaged activity (across all conditions) concurred with active electrodes defined using this method (see Figure 2).

### 2.6 Multilevel modelling

To understand the role of neural oscillations in temporal binding during automatic imitation we performed linear mixed-effects models. Unlike other approaches multilevel models has the advantage of performing well even with unbalanced data (Baayen et al., 2008; Bagiella et al., 2000). We investigated the impact of categorical (congruency) and continuous (reaction times, neural oscillations) predictors on single-trial temporal binding values. Correctly answered single-trial motor-response-locked data were matched to behavioral outcomes. We measured temporal binding through the assessment of time estimation errors. This corresponded to the difference between the time estimated by the participants and the real interval between actions and tones (either 0.3, 0.4 or 0.5 s). Larger negative values reflect heightened underestimation of time, and hence an increased temporal binding effect. To run the model, we selected as predictors neural oscillatory activities related to the responses elicited by the right hand (left alpha/mu, left beta, theta and gamma) as these may be directly related to motor responses. For each neural oscillation of interest, we extracted single-trial activities during active periods and we baseline-corrected on a trial basis. The average number of trials per subject was 66 (minimum: 35, maximum: 80) across congruency conditions. Single-trial temporal binding values were predicted from congruency (coded as 1/-1), reaction times (continuous), neural oscillatory activity (continuous) and delay (0.3, 0.4 and 0.5 sec; categorical) as fixed effects. Participants were modelled as random intercepts and random slopes. Neural activity, reaction times and temporal binding scores were standardized (z-scores) within participants. To fit multilevel models we used the lme4 package (Bates et al., 2014) in R (R Core Team, 2015) and calculated parameter estimates (b) and their associated t-tests (t, p) using the Satterthwaite approximation for degrees of freedom (Kuznetsova et al., 2017). The magnitude of the effects was bootstrapped with 95% confidence intervals.

## 3 Results

Behavioral findings from this study have been reported in a previous publication (Vastano et al., 2020). For the sake of completeness, they will be briefly described here again.

### Performance is increased for congruent actions

In the automatic imitation task participants responded to an imperative stimulus that appeared between two fingers while one finger simultaneously moved (Figure 1). Reactions times were recorded as the latencies between the imperative stimulus presentation and the participants’ responses. There was a main effect of congruency on error rates (F(2, 54) = 35.53, p < .001). Accuracy was higher for the congruent condition (98.4%) followed by the baseline (97.1%) and the incongruent (87.5%) condition (all ps < .001). Similarly, there was a main effect of congruency on reaction times (F(2, 54) = 108.93, p < .001). Reaction times were fastest for the congruent (427 ± 63 ms, mean ± standard deviation) compared to the incongruent condition (493 ± 78 ms; p < .05), while a marginal difference was observed between congruent and baseline conditions (475 ± 70 ms; p = .06). Overall, performance of the participants is facilitated by congruent relative to the baseline and incongruent conditions.

### Temporal binding is increased for congruent actions

Following the response of the participants and a delay, the participants listened to a tone and estimated the time elapsed between their actions and the tone (time estimation task). Temporal binding was measured as the difference between the participants’ estimations and the real interval durations in this task (time judgements errors). Larger temporal compression reflects increased temporal binding. There was a main effect of congruency (F(2,54) = 3.18; p < .05). We observed that the congruent condition (−72 ± 81 ms) led to significantly reduced time judgment errors than the incongruent condition (−62 ± 74 ms; p < .05), while a marginally significant difference was observed between the congruent and the baseline (−61 ms ± 80 ms; p = 0.06) conditions. Overall, temporal binding is increased when actions are facilitated relative to incongruent actions.

### Beta activity predicts temporal binding

To study neural activity, we first investigated significant oscillatory changes during action execution. Using a cluster-based permutation test, we identified modulations in neural activity within specific time windows. Notably, we observed a desynchronization in the 8-12 Hz alpha/mu and 13-30 Hz beta bands during action execution across both left and right electrodes, from -300 to 300 ms relative to movement onset (see Figure 2 for topographical representations of these changes). A pre-stimulus period, during which the hand remained stationary, served as the baseline. Participants’ responses elicit a strong bilateral desynchronization of both alpha/mu and beta oscillations during automatic imitation. The topography of these effects suggest the involvement of primary motor cortices (Hari & Salmelin, 1997). In addition, we observed an increase in synchronization of theta during action execution between -200 to 200 ms (relative to response) at frontal electrodes. Finally, we observed an increase in synchronization of gamma during action execution between 50 to 300 ms (relative to response) at centro-parietal electrodes (see Figure 2).

**Figure 3:**
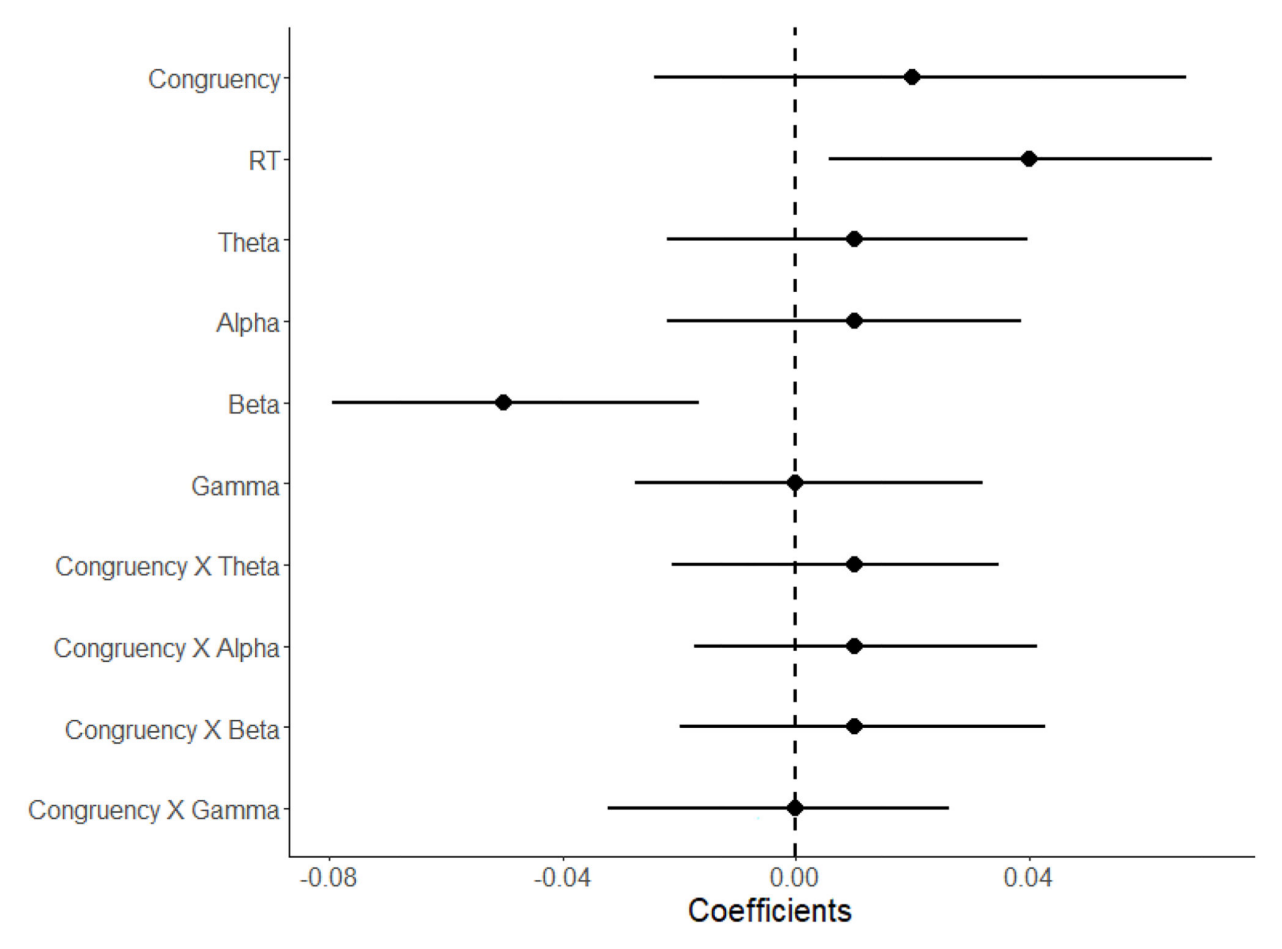
Model predicting temporal binding. The graph shows the estimated parameters and their confidence intervals for different predictor variables in the linear mixed model to predict temporal binding. The points represent the estimated values of the coefficients, while the vertical lines indicate the associated 95% bootstrapped confidence intervals. Note: The dashed horizontal point indicates zero value, and coefficients that do cross this point are not statistically significant (p > 0.05).

Next, we used a linear mixed-effects model to analyze the role of neural oscillations on temporal binding scores during the completion of the task. This is, single-trial temporal binding values were predicted from congruency (congruent, incongruent), reaction times and neural oscillatory activity (left alpha, left beta, theta and gamma power) as fixed effects. The temporal bindings values of the participants were modelled as random intercepts and random slopes. Our core finding was a main effect of beta power (b = -0.05, t(3640) = -3.1, p < .001, 95% CI = [-0.08 -0.01], see figure 3) on temporal binding scores. We also find an effect of reaction times (b = 0.04, t(3640) = 2.3, p < .05, 95% CI = [0.004, 0.07]) and an effect of delay (b = -0.5, t(3640) = -13.3, p < .001, 95% CI = [-0.6 -0.4]; not visualized in the figure but present in the model). No other main or interaction effects were found (see Table S1, in Supplementary Material to check the full model). We notice that a systematic effect of delay (greater underestimation with longer intervals) is an expected finding which converges with previous studies in temporal binding (Engbert et al. 2007; Vastano et al. 2017; Ulloa et al. 2019). The variation in delay between action and consequence is a necessary part of the experimental protocol to measure subjective time perception and thus, is considered a methodological variable to characterize the basic time estimation process and ensure variability. In this sense it is not a variable of interest and it will not be discussed further. Contrary to our expectations temporal binding was not modulated by congruency, and was not associated with changes in alpha/mu, theta or gamma oscillations. Our main finding is that the lower the beta desynchronization the larger the temporal binding effect. These results suggest that beta oscillations and thus the engagement of the motor system may underly the effects associated with temporal binding.

## 4 Discussion

Temporal binding is the compression of perceived time between an action and its outcome when a voluntary action produces a sensory event (Haggard, 2017; Haggard et al., 2002). Its very nature is linked to motor control models in which motor commands are coupled to predicted sensory consequences. Motivated by this motor basis, we assessed the impact of motor-related brain oscillatory activity and action congruency on temporal binding when participants engaged in an imitation-inhibition task. Our core finding is that trial-by-trial fluctuations in beta oscillatory activity predicted temporal binding scores: reduced beta desynchronization was associated with stronger temporal binding. This result suggests that motor beta dynamics are directly involved in shaping action-outcome temporal compression.

Beta (13–30 Hz) is a core rhythm of motor control (Pfurtscheller & Lopes da Silva, 1999). Beta decreases as a movement is prepared and executed, and then rebounds after movement, indexing motor state and evaluation (Neuper et al., 2006; Zaepffel et al., 2013). In our study, reduced beta activity (decreased beta suppression) during the response period predicted stronger temporal binding. One interpretation is a neural-efficiency account. When a movement is executed more fluently, the cortex can get by with less down-regulation of beta, so beta power is smaller. Prior work shows that with practice or expertise, sensorimotor activations and desynchronizations can shrink while performance improves (Babiloni et al., 2009, 2010; Koeneke et al., 2006; Li & Smith, 2021). On this view, the trials with tighter temporal binding are simply those with more efficient control. However, in our model we didn’t find any interaction of congruency with beta oscillations to predict temporal binding. If beta reflected movement difficulty or fluency, we would expect such an interaction. The absence of a congruency effect on temporal binding appears at odds with prior evidence that action fluency influences related constructs, such as judgments of agency (Chambon et al., 2014; Sidarus et al., 2017). Accordingly, interpretations invoking neural efficiency should be advanced with caution.

Another interpretation more consistent with the theoretical stance of this paper is a predictive account. The predictive coding framework proposes that the brain continuously generates predictions about sensory consequences of actions and updates these predictions based on actual sensory input (Friston & Kiebel, 2009; Shipp et al., 2013; Von Helmholtz, 1867). From this perspective, beta oscillations within M1 might reflect motor-level predictions about sensory outcomes arising directly from executed movements (Barone & Rossiter, 2021; Betti et al., 2021; Engel & Fries, 2010). Reduced beta suppression in M1 could indicate higher predictive precision at the motor execution level, decreasing the need for ongoing motor adjustments, a more stable motor output and then a stronger temporal binding between action and outcome. The lack of congruency modulation on beta further reinforces that beta’s association with temporal binding may be independent of transient motor conflict. One caveat of our approach was the inability to test another relevant brain activity related to motor processing: PMBR. With our design we couldn’t estimate it cleanly. The outcome tone and the speeded rating occurred too soon after the response, leaving little uncontaminated post-movement activity. We therefore found no reliable PMBR and focused on movement-related beta desynchronization, which our data sampled well. Overall, our findings are consistent with beta oscillations reflecting precision-weighted motor predictions that tighten perceived action–outcome coupling, independent of congruency. PMBR remains a question for future work.

We found no effect of congruency, nor congruency by oscillations interaction on temporal binding. The lack of congruency effect is an elusive issue. Because this is a re-analysis of the same dataset as Vastano et al. (2020), the divergence from earlier reports most likely reflects analytic specification, not task differences. Our trial-wise LME jointly estimates fixed and random effects, which improves the fidelity of EEG– behavior coupling estimates (Volpert-Esmond et al., 2021). We use partial pooling to stabilize trial-level effects (Gelman & Hill, 2006), include RT and other covariates to control confounds, allow random slopes to capture subject-specific coupling (Barr, 2013) and model a signed interval-error outcome to preserve directionality. These choices can attenuate small between-condition contrasts while emphasizing within-condition EEG–behavior coupling. Lastly, using single-trial EEG to track moment-to-moment neural fluctuations of subjective timing is consistent with the demonstrated relevance of ongoing slow activity shaping motor readiness and action timing (Jo et al., 2013, 2014; Schurger et al., 2012). Taken together, congruency is not ruled out, but trial-level neural variability better explains when action and outcome feel closer in time. Future work should replicate this with designs and analyses that capture trial-by-trial dynamics, ideally using diverse experimental approaches.

As a related observation, our LME revealed a trial-level association between reaction times and temporal binding: faster trials showed stronger temporal compression. We view this as informative but orthogonal to our main question on neural mechanisms. Critically, congruency did not affect temporal binding, so this seems not to be a congruency-driven facilitation. Instead, the main effect of reaction times points to a nonspecific facilitation/readiness factor: when responses are executed more efficiently, temporal binding tends to be stronger (regardless of congruency). More importantly, when reaction times and its interaction with neural oscillations were added to the model, the effect of beta activity on temporal binding remained essentially unchanged and the interaction between reaction times and beta was not significant (see Table S2, in Supplementary Material). Thus, motor beta activity appears to index a sensorimotor-predictive process that explains variance beyond simple speed. Reaction times has a main effect, but it neither mediates nor moderates the neural association. Both influences can coexist, with beta accounting for temporal binding variability that RT alone does not. This warrants future work that explicitly dissociates response speed from selection fluency and sensorimotor prediction.

We found no association between temporal binding and alpha/mu, theta or gamma power. These null results should be read cautiously: theta and gamma oscillations analyses were exploratory, but their absence suggests that conflict-monitoring and high-frequency sensorimotor encoding are not key drivers of temporal binding in this task. Among the two canonical sensorimotor candidates, mu/alpha suppression did not predict temporal binding. Movement-related beta remained the most informative signal in a relatively simple task (similar to Venot et al., 2024), tentatively favoring an important contribution of motor prediction to the trial-by-trial variance in temporal binding.

Several limitations qualify our findings and point to next steps. First, the association between beta power and temporal binding may reflect processes beyond motor execution (e.g., prediction or conflict); adjudicating among these possibilities will require designs that orthogonalize fluency, prediction and conflict, so that each can be estimated independently. Second, the present paradigm does not dissociate motor from sensory contributions to temporal binding. Factorial manipulations that independently vary motor predictability (e.g., variability in action plans or efference-copy reliability) and sensory predictability (e.g., outcome probability or latency) would clarify their relative weights in shaping temporal binding. Third, the study was not configured to quantify PMBR. Follow-up work should delay outcomes and ratings to secure an uncontaminated post-movement window and test whether PMBR predicts temporal binding. Fourth, temporal binding likely arises from a broader circuit that includes the cerebellum, supporting forward prediction and sub-second timing (Ito, 2008; Merchant et al., 2013; Miall & Wolpert, 1996) and basal ganglia–thalamo-cortical loops, which set motor state and temporal precision and support interval timing (Alexander et al., 1986; Matell & Meck, 2004; Middleton & Strick, 2000). Fifth, a key limitation is the absence of explicit, subjective agency measures. Future work should pair temporal binding with trial-level ratings of perceived control/causality to test whether the observed effects (and their link to movement-related beta desynchronization) reflect agency per se (implicit and/or explicit) rather than general temporal-perception bias.

In sum, our findings suggest that during voluntary action, trial-by-trial motor-beta activity relates to how much time compression is reported. This is consistent with the idea that motor-system dynamics shape the temporal alignment of action and outcome and contribute to the temporal binding phenomenon.

## Supplementary Material

Supplementary Material

## Highlights

- Temporal binding reflects the perceived temporal compression between voluntary actions and their effects.
- We used EEG and an automatic imitation task to examine how motor oscillations predict temporal binding.
- Reduced beta desynchronization was associated with stronger temporal binding at the single-trial level.
- Our findings suggest that beta oscillations contribute to action-effect coupling, possibly via motor-level predictive processes.
