# Supplementary material for: Motor beta oscillations contribute to the temporal binding effect
Source: Brain Cogn. Author manuscript; Available in PMC 2026 Jun 15. (PMC7619169; doi:10.1016/j.bandc.2025.106362)

**Motor beta oscillations contribute to the temporal binding effect**

Supplementary material

**Table S1.** Fixed effects from the linear mixed-effects model predicting temporal binding. Columns report the term, estimate, standard error (SE), degrees of freedom (DF), t statistic, and p value. Effects are considered significant at α = 0.05 (p < 0.05).


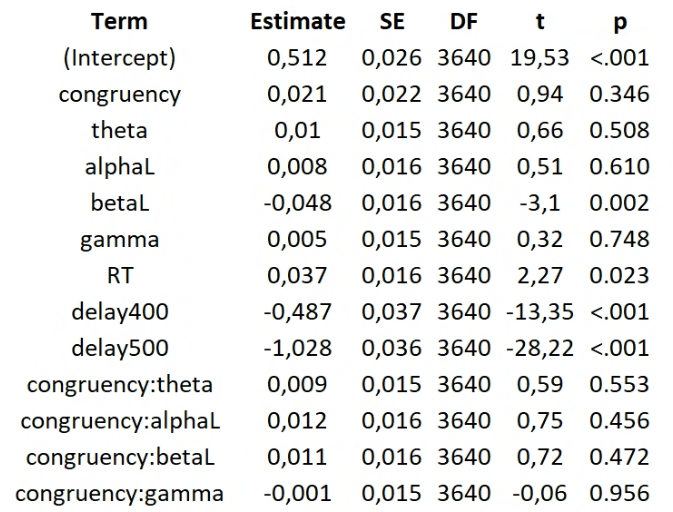


**Table S2.** Fixed effects from the linear mixed-effects model predicting temporal binding. Relative to Table S1, this model adds RT × oscillation interaction terms. Columns report the term, estimate, standard error (SE), degrees of freedom (DF), t statistic, and p value. Effects are considered significant at α = 0.05 (p < 0.05).


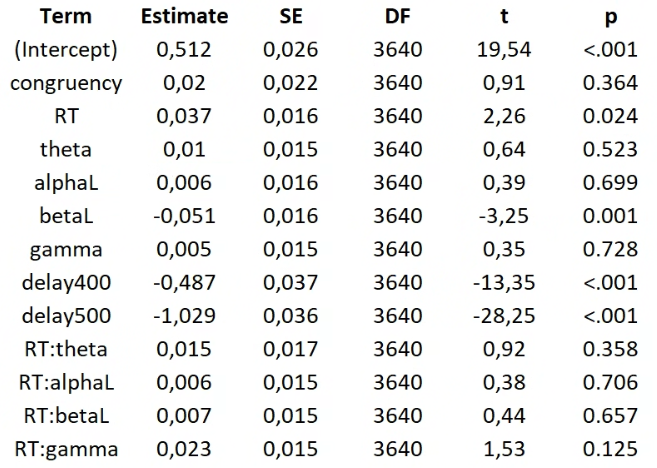

Supplement: Supplementary Material [file EMS214147-supplement-Supplementary_Material.docx]
